# Transposition and Intermingling of Gαi2 and Gαo Afferences into Single Vomeronasal Glomeruli in the Madagascan Lesser Tenrec *Echinops telfairi*

**DOI:** 10.1371/journal.pone.0008005

**Published:** 2009-11-24

**Authors:** Rodrigo Suárez, Aldo Villalón, Heinz Künzle, Jorge Mpodozis

**Affiliations:** 1 Escuela de Postgrado, Facultad de Medicina, Universidad de Chile, Santiago, Chile; 2 Departamento de Biología, Facultad de Ciencias, Universidad de Chile, Santiago, Chile; 3 Facultad de Ciencias de la Salud, Universidad Diego Portales, Santiago, Chile; 4 Anatomische Anstalt, Ludwig-Maximilians Universität, München, Germany; University of Lethbridge, Canada

## Abstract

The vomeronasal system (VNS) mediates pheromonal communication in mammals. From the vomeronasal organ, two populations of sensory neurons, expressing either Gαi2 or Gαo proteins, send projections that end in glomeruli distributed either at the rostral or caudal half of the accessory olfactory bulb (AOB), respectively. Neurons at the AOB contact glomeruli of a single subpopulation. The dichotomic segregation of AOB glomeruli has been described in opossums, rodents and rabbits, while Primates and Laurasiatheres present the Gαi2-pathway only, or none at all (such as apes, some bats and aquatic species). We studied the AOB of the Madagascan lesser tenrec *Echinops telfairi* (Afrotheria: Afrosoricida) and found that Gαi2 and Gαo proteins are expressed in rostral and caudal glomeruli, respectively. However, the segregation of vomeronasal glomeruli at the AOB is not exclusive, as both pathways contained some glomeruli transposed into the adjoining subdomain. Moreover, some glomeruli seem to contain intermingled afferences from both pathways. Both the transposition and heterogeneity of vomeronasal afferences are features, to our knowledge, never reported before. The organization of AOB glomeruli suggests that synaptic integration might occur at the glomerular layer. Whether intrinsic AOB neurons may make synaptic contact with axon terminals of both subpopulations is an interesting possibility that would expand our understanding about the integration of vomeronasal pathways.

## Introduction

In most mammals, the establishment and maintenance of social and sexual behaviours depend on the detection of semiochemicals by the vomeronasal system (VNS). The sensory surface of the VNS is the vomeronasal organ, a blind-ended tubular structure located bilaterally at the base of the nasal septum. Its neuroepithelium contains two spatially segregated populations of sensory neurons, each co-expressing either V1R receptors and Gαi2 protein or V2R receptors and Gαo protein, that send projections to distinct portions of the accessory olfactory bulb (AOB). Gαi2-expressing axons end in glomeruli located exclusively in the rostral half of the AOB, while Gαo-axons end in glomeruli of the caudal half of the AOB [1], [2], [3], [4], [5], [6], [7]. The dichotomic segregation of vomeronasal pathways into the AOB has been described in opossums [1], rabbits [8] and rodents [4], [9], [10], and was initially thought to represent a common feature of the mammalian VNS [11]. However, later reports showed that the V2R-Gαo pathway was absent in primates, shrews, goats, cows, horses, and dogs [12], [13], [14], [15].

In rodents, each pathway has been related to different functional specializations. Small, volatile ligands activate V1R-Gαi2 neurons [16], [17], [18], while responses to larger molecules, such as major urinary proteins, exocrine-gland secreted peptides and MHC class I peptides, have been recorded at V2R-Gαo neurons [19], [20]. In addition, stimulation with opposite-sex semiochemicals, in both males and females, activates more neurons of the rostral than the caudal AOB [21], [22], [23], [24], [25].

Synaptic integration of the segregated pathways has been shown to occur at two levels in the VNS. First, although mitral/tufted (M/T) cells contact 2–5 glomeruli of the same subdomain only [26], [27], [28], [29], they extend lateral dendrites that span through both subdomains [26], [27], [29]. Second, rostral and caudal M/T cells send projections that overlap, depending on the species, in the majority [30], [31], or the totality [32] of their recipient nuclei. As far as we know, the exclusive segregation of vomeronasal afferences occurs in all species with a two-pathway VNS, and no study has suggested that glomeruli of both pathways may undergo synaptic integration by intrinsic AOB neurons.

The objective of this study was to gain insights about the organization of vomeronasal afferences into the AOB of an early-branched placental mammal. We studied cellular organization and glomerular segregation at the AOB of the Madagascan lesser tenrec *Echinops telfairi*, (Afrotheria: Afrosoricida), a small species with insectivorous, nocturnal and solitary habits. This is the first time the vomeronasal pathways are investigated in the other great branch at the split of placental mammals. All previous studies in placentals have been done in Boreoeutherian species (superorders Euarchontoglires and Laurasiatheria). The ancestors of tenrecs branched off from the Boreoeutheria more than 100 million years ago [33], [34], [35]. We found several unprecedented traits that expand our knowledge about the neurobiology and evolution of the mammalian VNS.

## Results

### The AOB of the Lesser Tenrec, *E. telfairi*

**Figure 1:**
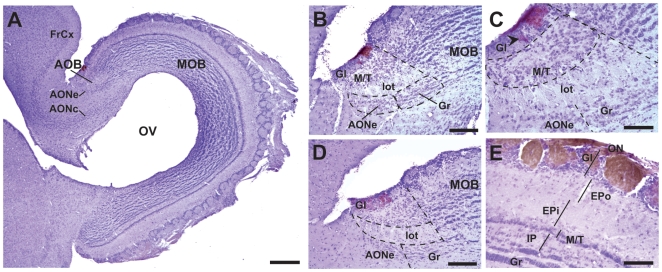
The accessory olfactory bulb (AOB) of the tenrec. Sagittal sections of the tenrec olfactory bulb immunolabelled against Gαi2 (A–C) or Gαo (D–E) proteins and counterstained with cresyl violet. (A) The relative sizes of the AOB and the MOB, and the continuity of the AONc towards the FrCx can be appreciated. Gαi2-expressing glomeruli are located at the rostral aspect of the AOB (A–C), however some glomeruli are displaced to caudal territories (arrowhead in C). The margin between the AOB and MOB is depicted by a discontinuous line (B–D). The Gr and AONe are ventral to the lot, and differ in their clustering (B). (C) Higher magnification of (B) showing the rostro-caudal asymmetry of M/T. (D) Gαo-expressing glomeruli are located at the caudal AOB, but not all caudal glomeruli seem to show full expression. (E) The cellular layers of the MOB are compactly stratified. All MOB glomeruli express Gαo protein, and they are larger and better defined than those of the AOB (compare with C, same magnification). AONc, anterior olfactory nucleus, central aspect; AONe, anterior olfactory nucleus external aspect; EPi, external plexiform layer, inner sublayer; EPo, external plexiform layer, outer sublayer; Gl, glomerular layer; Gr, granular cell layer; IP, internal plexiform layer; lot, lateral olfactory tract; M/T, mitral/tufted cell layer. Scale bar: 500 µm in A, 200 µm in B and D, and 100 µm in C and E.

As previously described [36], the olfactory bulbs of the tenrec are relatively large structures located at the rostralmost telencephalon (Figure 1). They contain a prominent olfactory ventricle (OV), surrounded by small and dense periventricular cells that may represent newly born neurons [37]. The AOB is located at the dorso-caudal extent of the main olfactory bulb (MOB) (Figure 1A). The anterior olfactory nuclear complex (AON), composed of an external (AONe) and a central (AONc) portion [36], partially encircles the deep MOB from its lateral aspect and can be observed lying below the AOB (Figure 1A). The AONe is a narrow band of densely packed cells, caudal to AOB granular cells, while the AONc prolongs caudally into the frontal cortex (FrCx), in a transition zone that has been referred to as the sulcal cortex [36]. There is a clear boundary between the MOB and AOB (dotted line; Figure 1B, 1C and 1D), marked by an abrupt discontinuity of MOB granular cells. Granular cells of the AOB lie below the lateral olfactory tract (lot; Figure 1C) and may be distinguished from AONe cells by their dense laminar packing and a more rostral distribution (dotted areas in Figure 1B).

Vomeronasal glomeruli expressing Gαi2 or Gαo proteins are arranged in rostral and caudal territories, respectively (Figure 1B and 1D). However, some Gαi2 glomeruli can be seen in caudal territories with biotinylated immunostaining (arrowhead in Figure 1C). The density and distribution of the mitral/tufted cell layer (M/T) is not homogeneous across the rostro-caudal axis of the AOB. It seemed more abundant and wider at its rostral extent, and consisted in 8–20 cells in depth (Figure 1C). In contrast, the M/T of the MOB is much narrower, containing up to 3 cells in depth (Figure 1E). The AOB seem to lack plexiform spaces, while the MOB has internal (IP) and external (EP) plexiform layers. The latter is divided in outer (EPo) and inner (EPi) sublayers that differ in cell density (Figure 1A and 1E). Glomeruli of the MOB are Gαo-positive, as also described in other mammals [10], [12], [38], [39] and are distributed in a compact layer of 1–2 glomeruli in depth (Figure 1E and Supporting information S1). They are larger than AOB glomeruli, and are individually surrounded by a large number of periglomerular cells (compare Figures 1C and 1E).

### Nonexclusive Segregation and Heterogeneity of Vomeronasal Glomeruli

**Figure 2:**
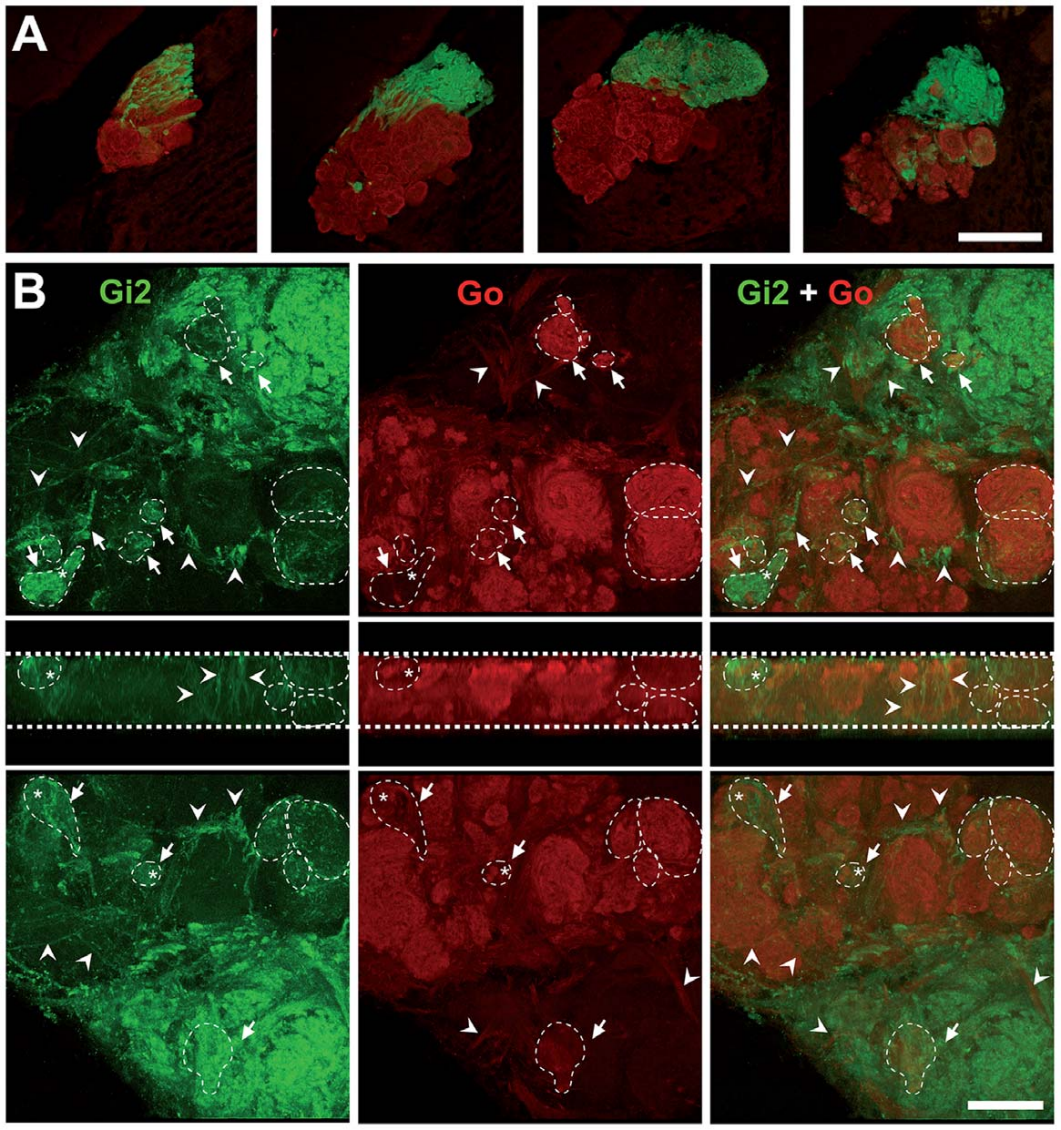
Non-exclusive segregation of vomeronasal afferences in the AOB of the tenrec. Confocal 3D reconstructions of Gαi2 and Gαo afferences into the AOB of a representative specimen. (A) Serial sagittal sections 50 µm width, separated 300 µm each, showing Gαi2-expressing axons (green) passing through Gαo-expressing glomeruli (red), to occupy an anterior position at the AOB. Sections are arranged from medial (left) to lateral (right). (B) Three-dimensional confocal reconstruction of the last picture of (A) represented as seen from one side (top row), from a ventral transverse view (middle row) and from the opposite side (bottom row). Each row shows Gαi2, Gαo and both labels. Arrows depict transposed glomeruli. Fibers that cross to the opposite subdivision are indicated with arrowheads. Interrupted lines contour glomeruli containing both Gαi2- and Gαo-expressing elements. Glomeruli presumably containing axonal boutons and/or neuropil of the adjoined subpopulation are indicated with asteriks. Scale bar: 200 µm in A, 50 µm in B.

All mammals with a two-pathway VNS studied so far show a clear-cut segregation of Gαi2 and Gαo positive glomeruli [4], [10], [27], [40]. In rodents, the vomeronasal nerve arrives from either the medial [27] or lateral [10] aspect of the olfactory bulbs, and bifurcates entirely into rostral and caudal territories before ending in glomerular neuropil [27]. In the tenrec, however, Gαi2-expressing axons arrive at the AOB passing through Gαo-expressing glomeruli to occupy a rostral position in more lateral sections (Figure 2A). Although vomeronasal glomeruli show a rostro-caudal segregation, some glomeruli of both populations locate within the adjacent subdomain (Figure 2A and 2B). The transposition of Gαi2 and Gαo-positive glomeruli into the adjacent subdomain was observed in all animals examined (n = 5). The relative position of transposed glomeruli was conserved across individuals (Supporting information S1).

A 50 µm three-dimensional confocal reconstruction of the last section of Figure 2A is shown at higher magnification in Figure 2B. The glomerular layer is displayed as if seen from one side (upper row), from ventral (middle row) and from the opposite side (lower row). Afferent axons from both populations, some of them with apparent varicosities, cross to the adjacent subdomain (arrowheads, Figure 2B) to end in transposed glomeruli (arrows, Figure 2B). Furthermore, and to our surprise, some glomeruli contained both Gαi2 and Gαo intermingled afferences (dotted regions, Figure 2B). Although decussating axons often passed between glomeruli, some entered into converse glomeruli and terminated in what seemed to be axonal boutons and/or glomerular neuropil (asterisks, Figure 2B). Fluorescent labeling of Gαo at MOB glomeruli also revealed corpuscular structures that may represent axonal endings (Supporting information S1). The organization and intermingling of afferences within single vomeronasal glomeruli can be better appreciated in an animated three-dimensional reconstruction of the same preparation (Video S1).

## Discussion

### Diversity in the Synaptic Organization of Chemosensory Systems

**Figure 3:**
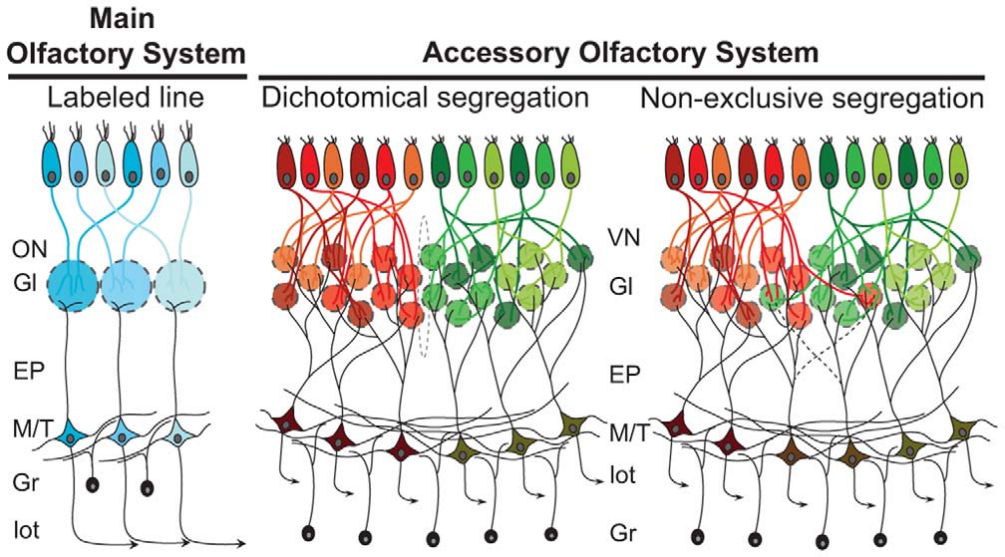
Patterns of connectivity in chemosensory systems. The organization of the main olfactory system has been regarded to as a labeled line. Sensory neurons expressing the same receptor converge their afferences into single glomeruli, where synaptic contact is established with only one M/T cell, in a one-to-one topology. Thus, each olfactory receptor is represented by a single M/T cell. The accessory olfactory system, however, is integrative in the sense that each sensory neuron sends projections to several glomeruli and each M/T cell contacts several glomeruli. Although M/T cells contact glomeruli receiving afferences from different receptors, they integrate glomeruli from the same V1R or V2R subpopulation, most probably from closely related receptors. This dichotomic segregation suggests that no M/T cell contact glomeruli from both subpopulations. The non-exclusive segregation of vomeronasal afferences of the tenrec is characterized by the integration of afferences from both subpopulations into single glomeruli, suggesting that AOB neurons (such as M/T or periglomerular) may also make synaptic contact with both subpopulations.

The organization of vomeronasal sensory representation at the AOB has revealed several differences when compared to the more-studied MOB. Main olfactory neurons expressing the same receptor converge their terminations into a single glomerulus, which is innervated by neuropil from a single apical dendrite of a M/T neuron [41]. Thus, a one-to-one topography is established between glomeruli expressing the same receptor and M/T cells, resembling a labeled line of sensory processing (Figure 3). In contrast, the synaptic organization between sensory afferences and M/T efferences at the AOB is integrative. While axons expressing the same vomeronasal receptor project to several glomeruli, each M/T cell may contact up to 5 glomeruli expressing the same [42], [43], or similar receptors [28], within a single subdomain. Moreover, each glomeruli is contacted by 1–3 M/T cells [27]. Thus, a high combination of synaptically integrated elements may converge onto individual M/T cells (Figure 3). The integration of glomeruli expressing different receptors occurs within each subdomain only and no study has reported, to our knowledge, evidence that any AOB neuron -projection or interneuron- would make synaptic contact with terminals from both subpopulations. Indeed, a space rich in glial cells and void of axonal connections, named linea alba, has been described at the margin between both AOB glomerular subpopulations of the rat [27]. Similarly, in the Caviomorph rodent *Octodon degus*, we described an invagination spanning all cellular layers at the margin between rostral and caudal territories [10], further supporting the notion of a structural and functional independence of both subdomains.

In the tenrec, however, we found that the segregation is not exclusive, as not only were some glomeruli located within the adjoining subdomain, but also some seem to receive mixed afferences from both populations (Figure 2 and Figure 3). These results suggest that individual AOB neurons may integrate synaptic activity from both vomeronasal pathways. To elucidate this, additional experiments, such as cell filling of AOB neurons combined with differential sensory immunolabeling, would be required.

Targeting of sensory axons, in both main and accessory olfactory systems, is directed by the specific expression of chemorepulsive peptides, mostly of the Semaphorin and Slit families, and their membrane receptors [44], [45]. Whether particular patterns of chemorepulsive molecules and their receptors are present in transposed and/or intermingled glomeruli deserves further investigation.

**Figure 4:**
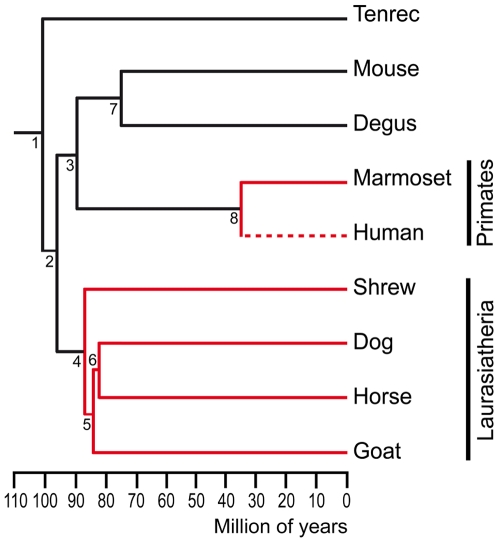
Distribution of the vomeronasal pathways in Eutheria. Chronogram of placental mammals showing the presence of both vomeronasal pathways (black lines), the V1R-Gαi pathway only (red lines), or the complete loss of both pathways (interrupted line). Two independent events of degeneration of the V2R-Gαo pathway might have occurred in the lineages leading to Primates and Laurasiatheria. Estimated times of divergence are based on refs. [33], [34], [35] for nodes 1–4, 6, 7, and on ref. [48] for node 5.

We have shown that, although not fully segregated, both vomeronasal pathways are present in the AOB of the tenrec. Results from comparative studies of Gα-protein expression at the VNS [12], [13] and genomic enquiries of functional V2R gene sequences [14], [15] lead to the parsimonious assumption that at least two events of deterioration of the V2R-Gαo pathway have occurred, independently, in the lineages leading to Primates and Laurasiatheria (Fig. 4). A possible scenario is that the Gαi2 pathway, which is conserved in all species with a functional VNS, would play a similar role in pheromonal communication across species, perhaps mediating the assessment of reproductive status between the sexes.

Whether alternative configurations to the dichotomical segregation of the vomeronasal pathways were present in early mammalian species is an interesting possibility that deserves further comparative analysis in early-branched placental and non-placental mammals.

## Materials and Methods

All experimental procedures followed the National Institute of Health Guide for the Care and Use of Laboratory Animals (NIH Publications No. 80–23, 1996) and were approved by the faculty ethics committee (Comité de Etica de la Facultad de Ciencias, Universidad de Chile). All efforts were made to minimize the number of animals used and their suffering.

We employed eight (3 females and 5 males) Madagascan lesser tenrecs (*Echinops telfairi*) raised in a breeding colony in Munich [46], weighing 66–153 g (110.5 g mean weight). The animals were deeply anesthetized (tribromoethanol, 1 ml/100 g body weight, ip.) and perfused as previously described [47]. We obtained 50 µm thick sagittal and coronal sections of the olfactory bulbs with a freezing microtome. Slices were either mounted for cresyl violet staining or collected in vials for further immunohistochemical processing.

Sagittal sections were incubated overnight in 3% normal goat serum (NGS) in PBS with 0.05% Triton X-100 (PBST) at 25°C. Then, they were incubated in primary immunoglobulins against Gαi2 (1∶200, mouse monoclonal, cat no. sc-13534, Santa Cruz Biotechnology, Santa Cruz, CA) and Gαo (1 µg/ml, rabbit polyclonal, cat no. 551, Medical and Biological Laboratories, Nagoya, Japan or 1∶200 mouse monoclonal, cat no. sc-13532, Santa Cruz Biotechnology, Santa Cruz, CA, for single or double reactions, respectively) with 3% NGS in PBST for 3 days at 25°C. The sections were then rinsed in PBS and incubated in a mixture of fluorescent goat anti-mouse and anti-rabbit secondary antibodies (1∶200; Alexa fluor 568 and 633 nm, respectively, Invitrogen), or in biotinylated goat anti-mouse antibodies (1∶200, cat no. sc-2039, Santa Cruz Biotechnology, Santa Cruz, CA) for 2 hours. Biotinylated sections were processed as described before [10]. They were mounted on gelatine-coated slides, counterstained with cresyl violet, observed under light microscopy (BX60; Olympus Optical, Thornwood, NY) and photographed with SPOT camera and software (Spot Advanced; Diagnostic instrument, Sterling Heights, MI). Fluorescent sections were examined with a confocal laser-scanning microscope (Zeiss LSM 510 Meta; Jena, Germany) using laser beams of 488 and 633 nm for excitation. Three-dimensional reconstructions and analyses were made with the LSM 510 software (version 3.2). All figures were prepared for presentation purposes with Adobe Photoshop CS3 (Adobe Systems, San Jose, CA).

## Supporting Information

Supporting Information S1 (0.05 MB PDF)

Video S1 Confocal reconstruction of Gαi2-positive axons (green) and Gαo-axons (red) in the AOB of the tenrec.
(9.67 MB MOV)
