# Supplementary material for: Transposition and Intermingling of Gαi2 and Gαo Afferences into Single Vomeronasal Glomeruli in the Madagascan Lesser Tenrec Echinops telfairi
Source: PLoS One. 2009 Nov 24;4(11):e8005. doi: 10.1371/journal.pone.0008005 (PMC2776991; doi:10.1371/journal.pone.0008005)
Supplement: Supporting Information S1 — (0.05 MB PDF) [file pone.0008005.s001.pdf]

Supporting Information S1 for “Transposition and Intermingling of Gαi2 and Gαo Afferences into Single Vomeronasal Glomeruli in the Madagascan lesser Tenrec *Echinops telfairi*” Rodrigo Suárez, Aldo Villalón, Heinz Künzle, Jorge Mpodozis.

Table S1. Animals used in this study and the presence of transposed glomeruli.

Animal	Sex	Gαi2 expression	Gαo expression	Transposed glomeruli	Detection method	Section plane
Et06-14	M	rAOB	cAOB, MOB	yes- Gαi2 and Gαo	biotin	sagittal
Et06-19	M	rAOB	cAOB, MOB	yes- Gαi2 and Gαo	biotin	sagittal
Et06-15	M	rAOB	cAOB, MOB	yes- Gαi2 and Gαo	biotin & fluorescence	sagittal
Et06-16	F	rAOB	cAOB, MOB	yes- Gαi2 and Gαo	biotin & fluorescence	sagittal
Et06-21	F	rAOB	cAOB, MOB	yes- Gαi2 and Gαo	biotin & fluorescence	sagittal
Et06-17	M	-	-	-		coronal
Et06-22	M	-	-	-		coronal
Et06-20	F	-	-	-		coronal

Localization of Gα-protein expression and the detection technique (biotin and/or fluorescence) used to observe transposition of AOB glomeruli. Coronal sections were not informative on glomerular transposition but helped to determine bulbar anatomy. M: male; F: female; rAOB: rostral accessory olfactory bulb glomeruli; cAOB: caudal accessory olfactory bulb glomeruli; MOB: main olfactory bulb glomeruli.

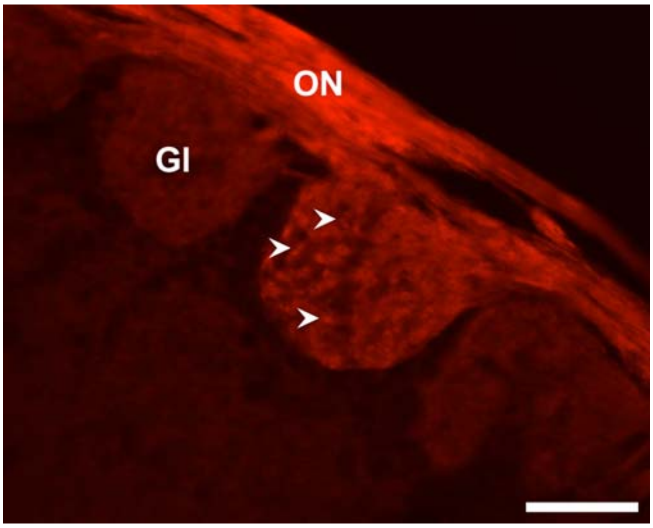

Figure S1. Fluorescent immunolabeling of Gαo-protein in the main olfactory bulb (MOB) of the tenrec.

The olfactory nerve (ON) and glomerular layer (Gl) of the main olfactory bulb show Gαo-protein expression. Some corpuscular boutons and/or neuropil can be seen within glomeruli (arrowheads). Scale bar: 100μm.

Other Attachments: Spanish abstract

Resumen

El sistema vomeronasal (VNS) de mamíferos participa en la comunicación socio-sexual entre conspecíficos mediada por feromonas. En el órgano vomeronasal, dos poblaciones de neuronas que expresan proteínas Gαi2 o Gαo envían proyecciones que terminan en glomérulos distribuidos en la zona rostral o caudal del bulbo olfatorio accesorio (AOB) respectivamente. Las neuronas de proyección del AOB, a su vez, contactan glomérulos de una misma subunidad. La segregación dicotómica de glomérulos del AOB ha sido descrita en zarigüeyas, roedores y conejos, mientras que Primates y Laurasiatherios perdieron la vía Gαo, o ambas (como en simios, algunos murciélagos y diversas especies acuáticas).

Estudiamos el AOB del tenrec malgache *Echinops telfairi* (Afrotheria: Afrosoricida), y encontramos expresión de las proteínas Gαi2 y Gαo en glomérulos rostrales y caudales respectivamente. Sin embargo, la segregación de estos glomérulos no es exclusiva, ya que ambas vías contienen algunos glomérulos translocados en la subdivisión contigua. Más aún, algunos glomérulos parecen contener aferencias mezcladas de ambas vías.

Tanto la transposición como la heterogeneidad de aferencias vomeronasales son rasgos, a nuestro saber, nunca antes descritos. La organización de glomérulos en el AOB del tenrec sugiere que puede haber integración sináptica ya en la capa glomerular. El que neuronas intrínsecas del AOB establezcan contacto sináptico con terminales axónicos de ambas subpoblaciones es una interesante posibilidad que expandería nuestro entendimiento sobre la integración de las vías vomeronasales en mamíferos.
